# Evaluating diversity and stereotypes amongst AI generated representations of healthcare providers

**DOI:** 10.3389/fdgth.2025.1537907

**Published:** 2025-04-25

**Authors:** Anjali Agrawal, Gauri Gupta, Anushri Agrawal, Himanshu Gupta

**Affiliations:** ^1^ Department of Computer Science, The University of Texas at Austin, Austin, TX, United States; ^2^ Ridgewood High School, Ridgewood, NJ, United States; ^3^ Rouse High School, Leander, TX, United States; ^4^ Heart and Vascular Institute, Valley Health System, Ridgewood, NJ, United States

**Keywords:** generative AI, sex diversity, race diversity, healthcare provider, stereotypes, DALL-E, Google Vision, machine learning

## Abstract

**Introduction:**

Generative artificial intelligence (AI) can simulate existing societal data, which led us to explore diversity and stereotypes among AI-generated representations of healthcare providers.

**Methods:**

We used DALL-E 3, a text-to-image generator, to generate 360 images from healthcare profession terms tagged with specific race and sex identifiers. These images were evaluated for sex and race diversity using consensus scoring. To explore stereotypes present in the images, we employed Google Vision to label objects, actions, and backgrounds in the images.

**Results:**

We found modest levels of sex diversity (3.2) and race diversity (2.8) on a 5-point scale, where 5 indicates maximum diversity. These findings align with existing workforce statistics, suggesting that Generative AI reflects real-world diversity patterns. The analysis of Google Vision image labels revealed sex and race-linked stereotypes related to appearance, facial expressions, and attire.

**Discussion:**

This study is the first of its kind to provide a ML-based framework for quantifying diversity and biases amongst generated AI images of healthcare providers. These insights can guide policy decisions involving the use of Generative AI in healthcare workforce training and recruitment.

## 1 Introduction

With only 5% of physicians identifying as African American and 36% identifying as females, there is a significant lack of diversity within the medical workforce (1–3). A diverse workforce is crucial for improving healthcare access and outcomes, particularly in underserved communities (4, 5). Yet, visual materials in medical education, workforce recruitment, and healthcare policy often mirror these disparities, inadvertently reinforcing existing inequities.

Recent advantages in text-to-image generative models, such as Dall-E, have enabled the rapid production of visual content, particularly in healthcare. Dall-E (6) is a generative AI system that can generate realistic images from text descriptions. It is trained on a large dataset of image-text pairings, allowing it to learn common visual features associated with certain terms (7). Despite the many applications of Dall-E, most current research on generative AI in healthcare has predominantly focused on large language models (LLMs) such as GPT-4, Gemini, etc. To illustrate, a PubMed query of “Dall-E” has only 87 results, while the query for “GPT” has 7,471 results. The few studies on Dall-E tend to explore the validity and appeal of generated images in a variety of contexts ranging from CPR illustrations to imagery on congenital heart diseases (8, 9).

Biases in generative AI systems—arising from training data, algorithms, and human decisions—can lead to the underrepresentation of minorities and the perpetuation of stereotypes. These biases raise ethical concerns regarding fairness, transparency, and accountability, as AI-generated content can influence public perception and clinical decision-making (10–13). There have been many attempts to mitigate biases in AI systems, such as resampling, fair representation, and optimized pre-processing of training data (14, 15). However, these techniques have been challenging to implement due to a lack of a unified definition of bias in the AI community; as a result, even new versions of AI tools that aim to mitigate prior concerns may continue to present biases.

As of now, there are few studies exploring gender and racial biases in Dall-E-generated images, and these too are in very narrowly defined contexts, such as images of medical imaging professionals, orthopedic surgeons, or pharmacists (16–18). A major limitation of these studies is that they all focus on only quantitative diversity metrics without considering qualitative dimensions of bias. In our study, we address this gap by employing Dall-E and Google Vision (19) to develop a novel methodology that assesses both quantitative (race, sex, and age diversity) and qualitative biases in AI-generated images of the healthcare workforce. Our study uniquely utilizes a more comprehensive diversity scale that accounts for the quality and realisticness of generated images. Additionally, we leverage computer vision tools to evaluate qualitative visual stereotypes. We hypothesize that Dall-E-generated images will not only reflect existing workforce disparities but may also reinforce gendered and racial stereotypes. By highlighting these ethical and social implications, our work aims to inform strategies for mitigating biases.

## 2 Methods

### 2.1 Image generation

**Figure 1:**
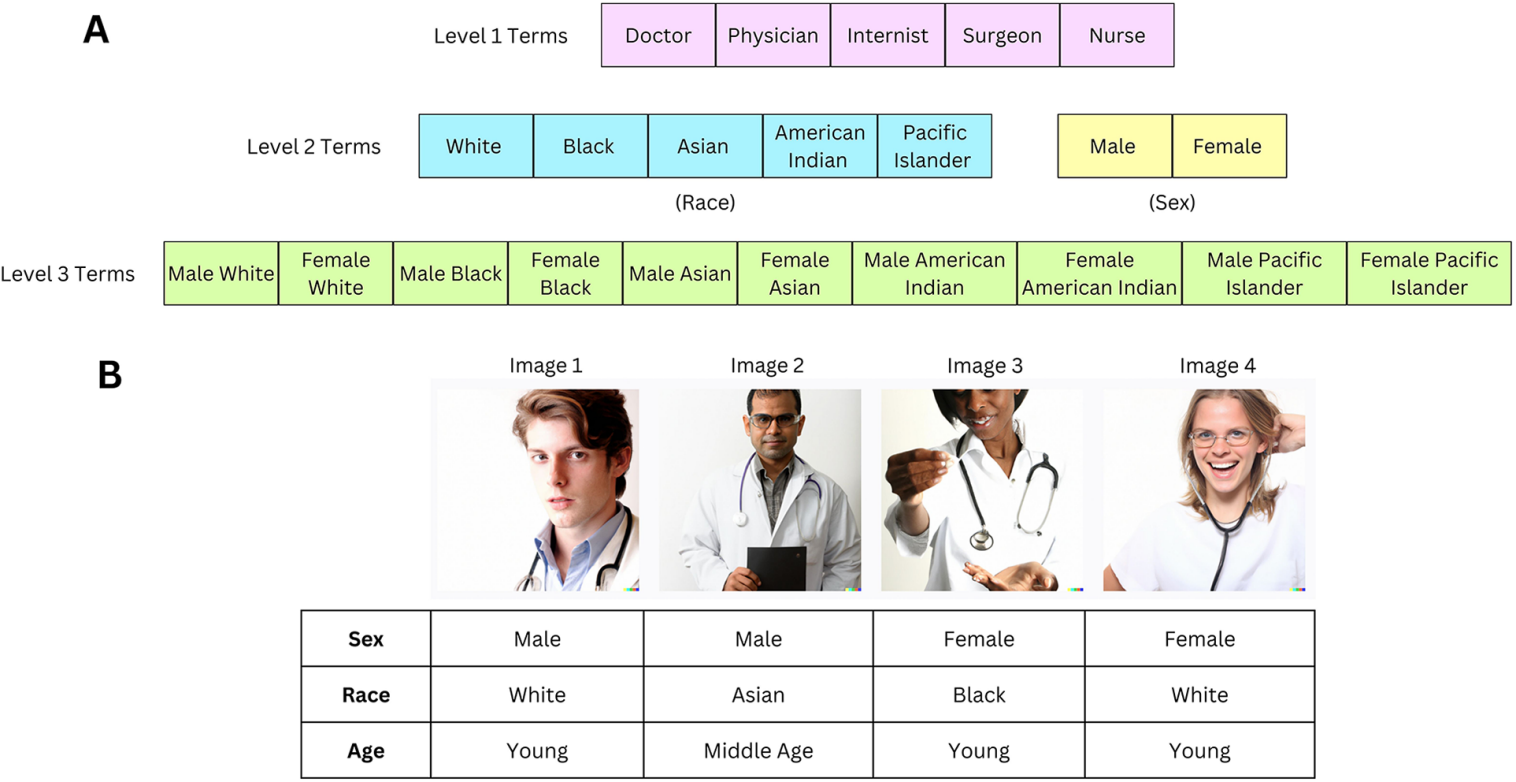
**(A)** Generic healthcare provider terms are listed in level 1 terms. One race or sex identifier is added to Level 1 terms to create Level 2 terms. Combinations of Level 2 terms (with both race and sex identifiers) create Level 3 terms. The full list of terms used to generate images is in Supplementary Table 8. **(B)** The Level 1 “Doctor” term generated the following image composite. Our assessment of sex, race, and age based on consensus scoring is described below each image in the table. This image was created with the assistance of DALL-E.

We used Dall-E, a text-to-image Generative AI tool, to generate synthetic images of healthcare providers, based on terms such as “doctor,” “physician,” “internist,” “surgeon,” and “nurse.” “Nurse” term was included to allow for a comparative analysis of diversity between doctor and nurse representations. Each term was further refined with specific race and sex identifiers (e.g., “Black doctor”, “female nurse”). The additional race identifiers selected were “Black,” “White,” “American Indian,” “Pacific Islander,” and “Asian,” based on U.S. Census bureau recommendations (20). The additional sex identifiers were “male” and “female” (21). A diagram illustrating the hierarchical labeling process to generate the terms can be found in Figure 1A, and the full set of terms is listed in Supplementary Table 8. For each term, 4 individual images were generated that were extracted as one composite, as shown in Figure 1B. A total of 90 composites consisting of 360 images were generated between December 2022 and July 2023.

As depicted in Figure 1A, Level 1 terms correspond to “doctor”, “physician”, “internist”, “surgeon”, and “nurse”. Level 2 terms correspond to the addition of either a race identifier or sex identifier to the Level 1 term (but not both types of identifiers) (e.g., “Black Doctor”, “Female Doctor”). Level 3 terms correspond to the addition of both race and sex identifiers on a Level 1 term (e.g., “Female Black Doctor”).

### 2.2 Quality and realisticness assessment of images

We performed an initial assessment of the quality and realisticness of the generated images. Each image was evaluated on three criteria:

1. **Image Quality**: Whether the image displayed a complete or partial face.
2. **Realisticness**: Distortion in facial features (blurriness, uneven features) or color discrepancies.
3. **Clipart Content**: The presence of clipart (graphics) in the generated images was noted, with a range of 0–4 individuals flagged as clipart per composite.
4. See Supplementary Material for additional details.

### 2.3 Qualitative assessment of diversity

We evaluated sex, race, and age diversity for each composite consisting of 4 images. The composites were rated on a scale from 1 to 5 for sex and race diversity and 1–3 for age diversity based on consensus scoring (AA, AA, GG). 5 (or 3 for age diversity) represents maximum diversity (e.g., equal representation of females and males) while 1 represents least diversity (e.g., all 4 images are males)

1. Sex Diversity (1–5)—a measure of the apparent representation of female and male sexes
2. Race Diversity (1–5)—a measure of the apparent representation of different races
3. Age Diversity (1–3)—a measure of the apparent representation of different age groups

If the search term included a race or sex identifier, race or sex diversity, respectively, was not evaluated. A full explanation of our scale for each of these following factors can be found in the Supplement and Supplementary Tables 1–3. Our scale acknowledges the inclusion of intersex individuals (where the sex was ambiguous) and multiracial individuals in the images. Additionally, we provide an example rating of the term “Doctor” in Figure 1B.

### 2.4 Validation of consensus scoring and diversity scale

The validity of the consensus scoring method was evaluated using the Python package DeepFace (22), a deep learning-based facial recognition system. DeepFace was employed to detect the sex, race, and age of the same terms for which consensus scoring was used to assess sex and race diversity. During consensus scoring, we had recorded age in the following buckets—middle-aged, elderly, young. For validation with DeepFace, which returns an exact age, middle-aged corresponds to ages 35–50, young 20–35, and elderly 50+. However, DeepFace is not fully accurate as it often failed to recognize all the faces in the original composite of four images, due to partial cropping of some faces or biases in DeepFace's algorithm. For the faces that DeepFace successfully recognized, we calculated the proportion of instances where the sex, race, and age bucket identified during consensus scoring matched that were predicted by the DeepFace algorithm. A consistency rate of ≥70% validated the consensus scoring methodology.

### 2.5 ML-driven image labeling and categorization

Google Vision is a tool that utilizes computer vision algorithms to assign labels and identify objects within images. In order to identify stereotypical associations present in the images, we used Google Vision to assign the top 10 labels for each image. Each label is associated with a confidence score, which we then normalized using the following formula:

$$\mathrm{Normalized}\;\mathrm{score}=\frac{\mathrm{score}-\min}{\max-\min}$$

where min is the minimum score amongst all labels, and max is the maximum score amongst all labels.

We categorized the labels assigned by Google Vision into 12 self-defined categories, such as “Clothing”, “Facial Expression”, and “Medical Tools” (see Table 1 for the full list). The categories were defined after examining the top labels assigned to images by Google Vision. The full set of labels, along with its normalized score and category, can be found in Supplementary Table 9.

**Table 1:** Category list.

Category	Definition	Top terms
Facial expression	Terms related to emotions and the act of expressing such emotions.	1. Smile 2. Facial Expression 3. Happy
Clothing (formal)	Clothing that is formal (typically associated with a professional setting) but is not Medical Clothing.	1. Collar 2. Dress Shirt 3. Workwear
Clothing (general)	Clothing that is not formal nor medical clothing (includes clothing for everyday use).	1. Sleeve 2. Outerwear 3. Clothing
Medical clothing	Clothing specifically worn by professionals working in healthcare.	1. White Coat 2. Scrubs 3. Protective Personal Equipment
Medicine	All medical terms that were not Medical Tools or Medical Clothing.	1. Service 2. Health Care Provider 3. White-Collar Worker
Medical tools	Instruments used by medical professionals that detect, prevent, and treat illnesses.	1. Stethoscope 2. Scrubs
Fashion occupation	Terms related to the field of fashion.	1. Fashion Design 2. Fashion Accessory 3. Jewellery
Color	Colors, typically characterizing colors in the image.	1. Electric blue 2. White 3. Azure
Body part	Internal and external components of the human body excluding facial features.	1. Neck 2. Shoulder 3. Joint
Miscellaneous	Any labels that did not belong in the other categories.	1. Event 2. Product 3. Photograph
Facial feature	Body parts that reside on the face.	1. Jaw 2. Eyelash 3. Eyebrow
Actions	Activity or movement done by a human.	1. Gesture 2. Standing 3. Animation

Description of all categories used to categorize the Google Vision labels assigned to images.

### 2.6 Clustering analysis

In this analysis, we examined 3 separate image cohorts. These cohorts were selected as we identified a significant difference between their categorical distributions, as shown in Table 2:

1. Images with “White” or “Black” identifier
2. Images with “White” or “Asian” identifier
3. Images with “Female” or “Male” Identifier

**Table 2:** Comparison of categorical distribution.

Cohort A	Cohort B	*P*-value
Male	Female	<0.001
White	Asian	0.035
White	Black	<0.01
White	American Indian	<0.01
White	Pacific Islander	0.79

Comparison of the overall distribution of assigned categories for generated images of different sex and race cohorts. A Chi Square test was used.

The objective of the clustering analysis was to group all images within each cohort according to the assigned categories of image labels. We used the KMeans algorithm from the *sklearn* Python library as our clustering method. K-means clusters similar data points by minimizing the distance between data points in the same cluster. We utilized Euclidean Distance to determine the distance between two images.

Formula for Euclidean distance between Image A and B = *d*(*p*, *q*) = $\sqrt{\sum_{i=1}^{n}{\left(q_{i}-p_{i}\right)}^{2}}$ where *q* represents the sum of normalized scores for all labels for a specific category in image A and *p* represents the sum of normalized scores for all labels for the same category in image B. *n* represents the total number of categories, which is 12.

For each cohort, the Elbow method heuristic was used to determine the optimal number of clusters. This heuristic involves plotting the within-cluster variance against the number of clusters, allowing us to identify the “elbow” point on the graph. The “elbow” point on the graph indicates the number of clusters where adding more clusters yields diminishing returns in reducing variance. We then ran Kmeans with this identified optimal number of clusters and recorded the names of the images classified in each cluster.

Within each of the three clustered image cohorts, we had included images from two separate groups: “White” vs. “Black”, “White” vs. “Asian”, and “Female” vs. “Male”. We then aimed to evaluate whether the images from one group were more dispersed across clusters than images from the other group. A greater dispersion of images from a group across clusters may suggest higher heterogeneity, or diversity, in the images. In contrast, if a group's images are more concentrated within a limited number of clusters, this could signify more homogeneity, or less diversity, in the images.

The entropy metric was used to determine the distribution of an image group (e.g., “Black”, “White”, “Male” images, etc.) across clusters.

$$\mathrm{Entropy}\;\mathrm{of}\;\mathrm{Group}\;A=\sum_{i=1}^{k}\;p(x_{i})\;*\;\log\;(\;p(x_{i}))\;*\;n_{i}\;$$

*k* is the number of clusters. $p(x_{i})$ is the proportion of images in cluster *i* corresponding to Group A. $n_{i}$ is the number of images in cluster *i*.

Higher values of entropy indicate a more diverse cluster distribution of images of that group.

We also recorded the most important category for each cluster as the one with the lowest average Euclidean distance to the cluster center for all points in the cluster. We recorded whether the average value for this category within the cluster was higher, lower, or approximately the same compared to the mean category value across all clusters.

### 2.7 Heatmaps of categorical distributions

For all images in a specific race/sex cohort, we calculated the proportion of labels assigned to each category. These proportions were used to create a categorical distribution for different cohorts, which were then depicted in heatmaps. One heatmap compares the categorical distribution of labels assigned to male vs. female images, and the other displays the distributions for all five race cohorts examined in this study.

### 2.8 Statistical analysis

The manual diversity ratings are on a 5-point or 3-point scale. When reporting the diversity ratings for a specific group/cohort of images, we converted this metric to a percentage out of the total possible points across images. A percentage of 100% (full diversity) indicates that each image on the cohort was rated 5/5 (for race/sex diversity) or 3/3 (for age diversity).

A one-sided z-proportion test was used to compare the proportion of labels assigned to a specific category between race/sex image cohorts (e.g., “Medical Clothing” category in “Females” vs. “Males”). One-sided z-proportion test was also used when comparing diversity between cohorts, after conversion to a percentage. A *p*-value < 0.05 was reported as statistically significant. To compare the entire categorical distribution of two image cohorts of a different race/sex (e.g., “Black” vs. “White”), a Chi-Square test was used.

## 3 Results

### 3.1 Validity of consensus scoring

The sex and race assigned through consensus scoring align with the classifications provided by DeepFace in 86.1%, 80.0%, and 78.6% of cases for sex, race, and age respectively (see Table 3 for full analysis). Causes for inconsistencies include the different race classification used by DeepFace (includes races such as “Indian” and “Middle Eastern” which are not present in our classification). Additionally, DeepFace is not fully accurate and has its own biases. Given this and the relatively high consistency, the diversity ratings from consensus scoring were considered valid.

**Table 3:** Deepface validation.

Metric	Value
Total images validated	160
Total images detected	103
Female match rate	71.0%
Male match rate	95.3%
Sex diversity match rate	86.1%
Race diversity match rate	80.0%
Age diversity match rate	78.6%

Distribution of Discrepancies Between Consensus Scoring and DeepFace Validation.

### 3.2 Qualitative assessment of diversity

**Figure 2:**
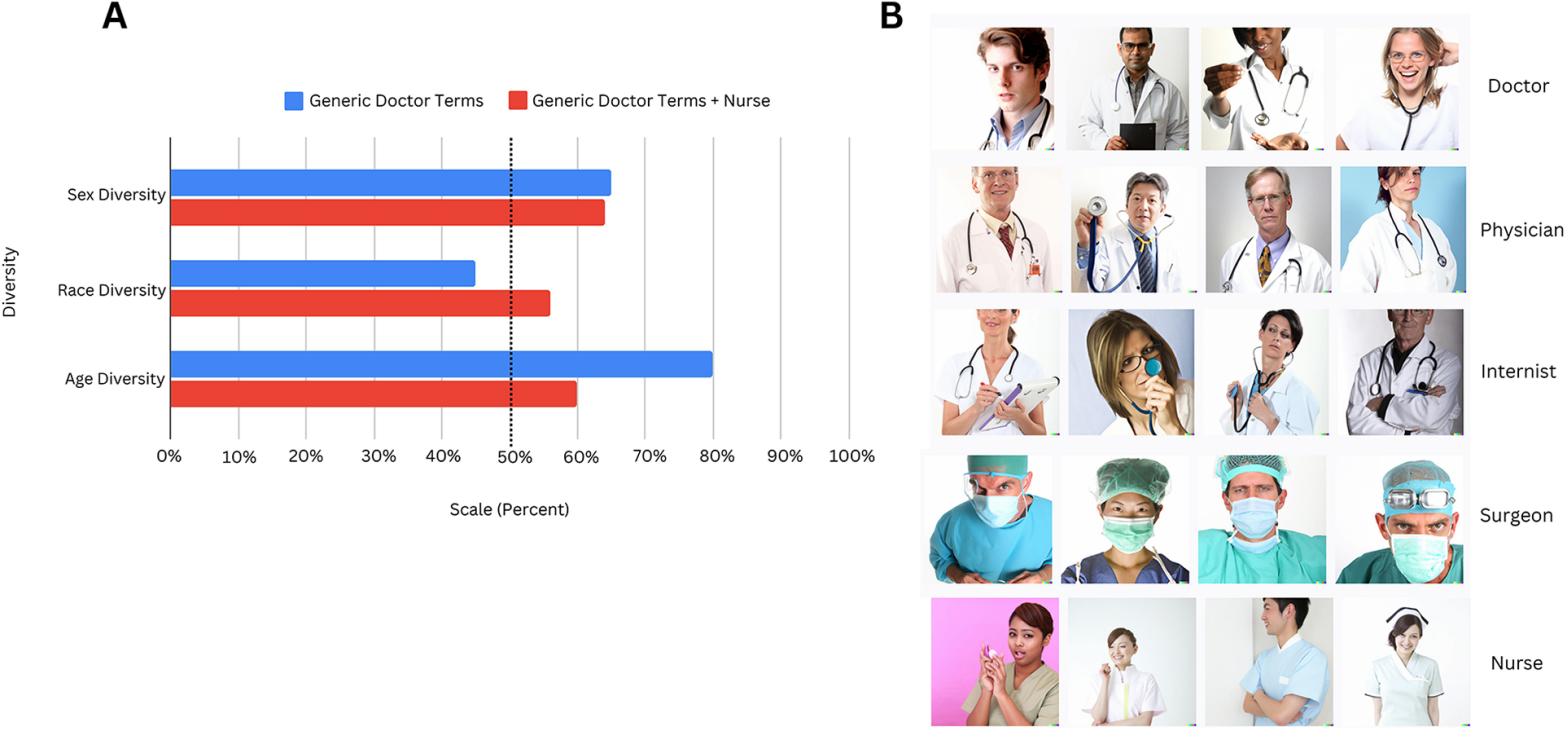
**(A)** Using Level 1 terms only, we describe the sex, race, and age diversity for two different cohorts—one with “Doctor,” “Physician,” “Internist,” and “Surgeon” (Generic Doctor Terms) and another with these 4 terms with the addition of “Nurse.” For Generic Doctor Terms alone, sex and race diversity, on a 5 point scale, was 3.25 and 2.25, respectively, and age diversity was 2.4 on a 3 point scale. For Generic Doctor Terms + Nurse, sex and race diversity was 3.2 and 2.8, respectively, and age diversity was 1.8. For comparison, the diversity metrics are represented as a percentage out of the maximum possible points that could be assigned. **(B)** Here, we present all the composites generated for the Level 1 terms that were used for the assessment of sex, race, and age diversity in Figure 2A. This image was created with the assistance of DALL-E.

We examined the sex, race, and age diversity amongst the images generated with level 1 terms only. The images corresponding to these terms are depicted in Figure 2B, and the diversity ratings in Figure 2A.

Figure 2A also compares the diversity ratings for two different cohorts to determine differences in the demographic representation of nurses and other healthcare providers:

Cohort 1—Images generated from terms “Doctor”, “Physician”, “Internist”, “Surgeon”

Cohort 2—Images generated from above terms + “Nurse”

Diversity was scored as a percentage of total possible points: For Cohort 2 images, sex diversity was 64%, race 56% (average scores of 3.2 and 2.8 out of 5, respectively), and age 60% (score of 1.8 out of 3). 100% represents maximum diversity. The cohort with “Nurse” has considerably lower age diversity than the cohort without “Nurse.”

We also compared diversity metrics between images of female and male healthcare providers using level 2 terms only (e.g., “Female Doctor”, “Female Physician”, etc. vs. “Male Doctor”, “Male Physician”, etc.). These results are in Supplementary Figure 1A, and we found that males had lower age diversity than females (*p* = 0.033). Diversity metrics were also compared between level 2 terms of healthcare providers of different race identifiers (Supplementary Figure 1B), although no significant differences could be found.

### 3.3 Clustering of differences between image cohorts

The full dataset on the specific images included in clusters for all tested cohorts is in Supplementary Table 10.

**Figure 3:**
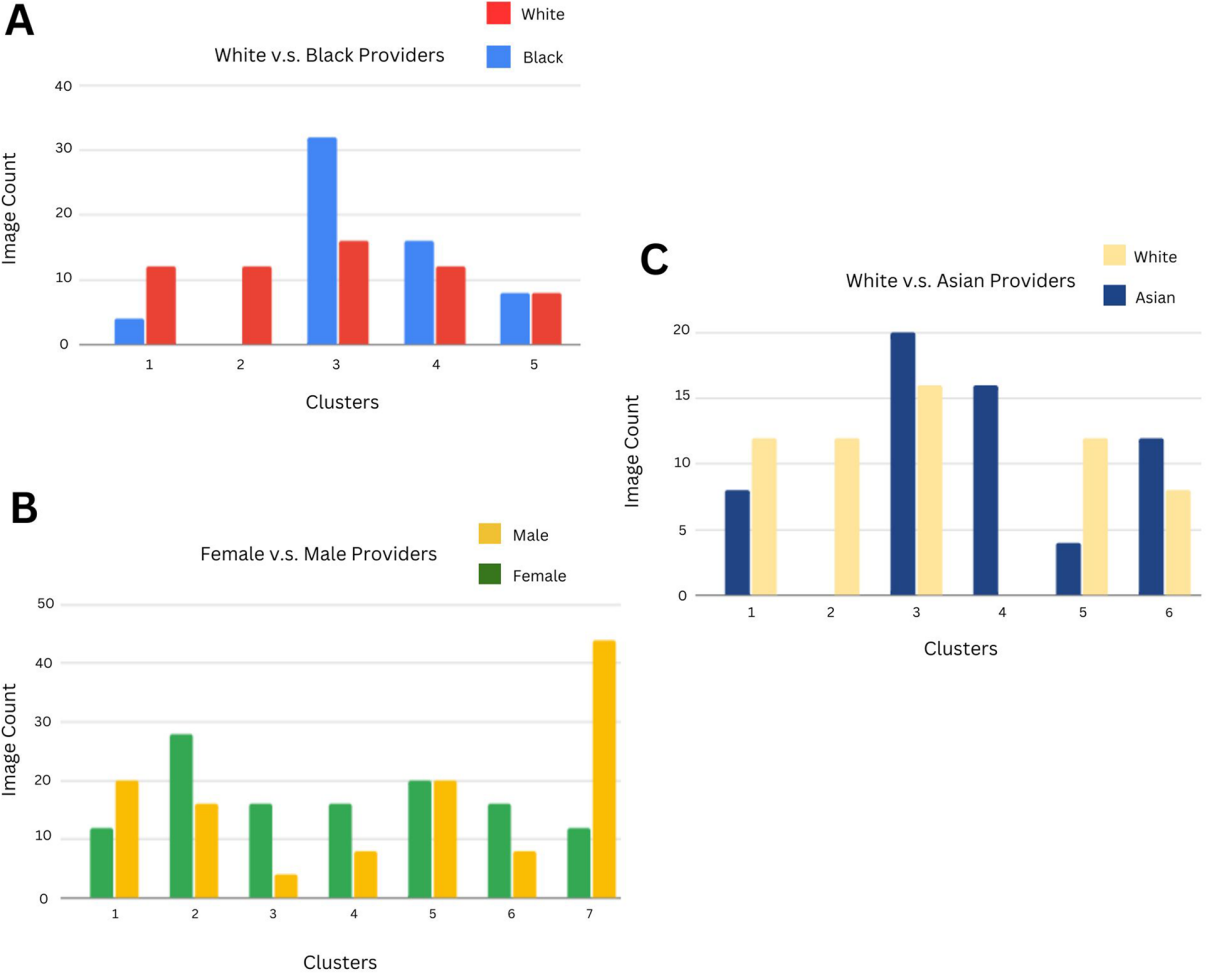
**(A)** Using a clustering methodology applied to Black and White Level 2 and 3 terms, we identified distinct clusters based on the categorical distribution of Google Vision labels. The count of Black vs White images in each cluster is indicated in each bar (which depicts a cluster). The primary category contributing to each cluster is written below, with ↑ denoting that the category's mean value is higher for that cluster than it is for other clusters, ↓ indicating that the category's mean value is comparatively lower for that cluster, and - indicating that the category's mean value is similar to that of other clusters. Cluster 1: Facial Expression ↓, Cluster 2: Formal Clothing ↓, Cluster 3: General Clothing ↑, Cluster 4: Medical Clothing -, Cluster 5: Medicine ↓. **(B)** Clustering methodology applied to Female and Male Level 2 and 3 terms. Cluster 1: Facial Expression ↓, Cluster 2: Formal Clothing ↓, Cluster 3: General Clothing ↓, Cluster 4: Medical Clothing ↓, Cluster 5: Medicine ↑, Cluster 6: Medical Tools ↓, Cluster 7: Fashion Occupation ↑. **(C)** Clustering methodology applied to Asian and White Level 2 and 3 terms. Cluster 1: Facial Expression ↓, Cluster 2: Formal Clothing ↓, Cluster 3: General Clothing ↑, Cluster 4: Medical Clothing ↑, Cluster 5: Medicine ↑, Cluster 6: Medical Tools ↓.

1. Image Cohort A—“White” vs. “Black” Images (Figure 3A)

Across 5 clusters, the entropy for White images was 3.99 and for Black Images 3.59, indicating that Black images were less distributed across clusters and hence more homogeneous. The largest cluster consisted of 32 Black (26.7% of all Black images) and 16 White images (13.3% of all White images), with general clothing as the most important category for this cluster.

2. Image Cohort B—“Male” vs. “Female” Images (Figure 3B)

Across 7 clusters, the entropy for Male images was 8.04 and for Female images 7.96, indicating a similar level of heterogeneity in Male and Female images. Three of the clusters with a higher proportion of female images (64%, 67%, and 67% of images were female, respectively) had lower mean values for the formal clothing, medical clothing, and medical tools categories.

3. Image Cohort C—“White” vs. “Asian” Images (Figure 3C)

Across 6 clusters, the entropy for Asian images was 3.34 and for White images 3.24, implying that Asian images were slightly more distributed across clusters than White images. For the homogeneous Asian cluster (consisting of 16 Asian images), the most important category was medical clothing. The category had a relatively high mean value of 0.19 as the average medical clothing category value across clusters was 0.066, implying that medical clothing labels were more predominant for Asian images when compared to White images.

### 3.4 Integrated assessment of stereotypes

**Figure 4:**
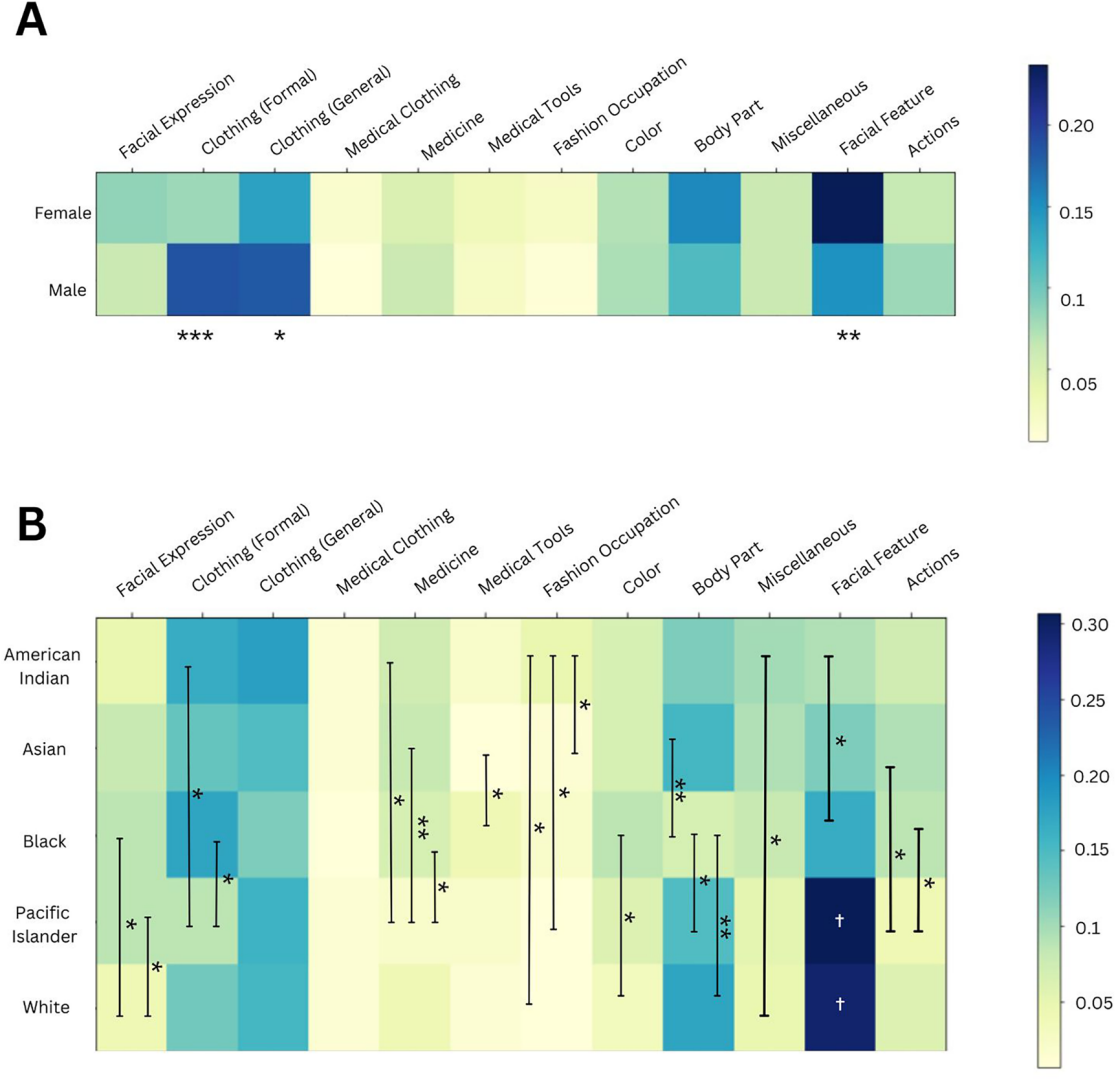
**(A)** Using Male and Female level 2 and level 3 terms, we compared the proportion of labels assigned to each category between these two cohorts, as depicted in the heatmap. Significant differences between male and female clothing and facial feature labels were identified. ***p-value <0.001; ** <0.01; and * <0.05. **(B)** Using Level 2 and Level 3 race terms, we compared the proportion of labels assigned to each category between these five race cohorts, as depicted in the heatmap. *** was used to signify a *p*-value <0.001, ** <0.01, and * <0.05. The white † in the facial feature column illustrates that Pacific Islander and White cohorts had significantly greater proportions of this category than all other racial cohorts.

Based on the categorical distribution of labels assigned to images of different cohorts, we noted differences in the image depictions of males vs. females (Figure 4A) and of different races (Figure 4B).

When comparing the categorical distribution for images of different sexes (Figure 4A), we found a smaller proportion of clothing-related labels in images of females compared to males, for both formal (***) and general clothing (*). Formal clothing includes “dress shirt” and “workwear” labels. Additionally, females had a higher proportion of facial feature labels, such as “eyelash” and “eyebrow”.

Similarly, there were differences in the categorical distributions for images of different racial groups (Figure 4B). White images had a smaller proportion of facial expression labels, when compared to “Black” and “Pacific Islander” images. This category of labels includes terms such as “smile” and “happy.” American Indian providers had the greatest proportion of fashion occupation labels, out of all racial groups, with these labels including “fashion design” and “fashion accessory.” Pacific Islander providers had the lowest proportion of formal clothing labels of all races.

We also compared the categorical distribution between different image cohorts using the Chi-Square test (Table 2). Male and Female images had significantly different distributions (***), along with White vs. Black or American Indian images (**). Interestingly, White and Asian images did have statistically different categorical distributions, but this was not as statistically significant as other group comparisons.

## 4 Discussion

In our study, we present a novel approach to evaluating biases in AI-generated images of healthcare professionals, specifically focusing on the Dall-E generative model. By analyzing race, sex, and age diversity, as well as visual stereotypes using Google Vision, we provide an empirical assessment of how generative AI systems reflect and potentially reinforce workforce disparities. Unlike prior studies that have primarily examined numerical racial and sex representation, our work extends to characterization of qualitative biases in images. While our approach is easily scalable, it also raises ethical concerns about AI transparency and misinterpretation of results.

Generative AI can provide unique insights into diversity as it leverages deep learning from very large real-world datasets (23)—a scale that humans cannot replicate in cross-sectional studies and surveys. The average composite score for sex diversity was 3.2 on a 5-point scale (or 64%) (Figure 2A), which aligns well with the report (24) that females constitute 37% of the physician workforce (or approximately 3.7 on our scale). A 100% score (5 points) is equivalent to a 50–50 distribution of females and males. Our race diversity score of 56% reflects real-world statistics (64% White, 5.7% Black) (24). Additionally, males had lower age diversity than females (Supplementary Figure 1A), consistent with the older age profiles of male-dominated specialties. These findings suggest that while Dall-E does not perfectly replicate real-world demographic distributions, it exhibits trends that approximate known disparities.

We used the Google Vision algorithm to assign labels to the images (19), and these labels were grouped into 12 self-defined categories. This methodology allows us to quantify the association between race and sex cohorts and specific categories, enabling us to measure stereotypes by identifying patterns in how certain objects or appearances are disproportionately attributed to particular groups (25).

We clustered different image cohorts (White vs. Black, White vs. Asian, and Male vs. Female) based on the distribution of these assigned categories (Figures 3A–C). Entropy quantified the spread of each race or sex image group across clusters, with higher entropy values indicating greater distribution of images across clusters. Black images showed 0.4 points lower entropy than White images, suggesting greater image homogeneity possibly due to stereotypical portrayals of Black healthcare providers. Male and female images had similar entropy levels, but in clusters with a female majority, mean category values for formal clothing, medical clothing, and medical tools were relatively lower when compared to other clusters. Additionally, Asian images had slightly higher entropy (0.1 points) than White images. One of the homogeneously Asian clusters had a greater prevalence of medical clothing labels, reinforcing the prevalent representation of this group in healthcare.

We also compared the categorical distribution of labels assigned to images of different race and sex cohorts using heatmaps (Figures 4A,B). For female healthcare provider images, we observed fewer formal clothing labels (e.g., “dress shirt”) compared to male images. This finding aligns with a prior study, which found that images of male Congress members were more frequently labeled with terms like “suit” and “tuxedo” by Google Vision (24). Additionally, female images contained more labels related to facial features. As the quality and realisticness were similar across both cohorts (Supplementary Figure 2A), this result cannot be attributed to variations in image cropping or distortion of facial features. The association of facial features with females projects stereotypical links between females and appearance, mirroring similar findings in the study on Google Vision labeling of images of Congress members (25).

Using Google Vision to evaluate categorical associations with race cohorts, we identified further stereotypes associated with Black, Pacific Islander, and American Indian healthcare providers. Black and Pacific Islander healthcare providers had disproportionately more facial expression labels, such as “smile”, than White providers (Figure 4B). Labeling images with “smile” may convey a less serious portrayal of Black providers (see Supplementary Figure 3 for a comparison of facial expressions in “Physician” images across races). A similar association with the “smile” label was noticed in female images of Congress members (25). Additionally, American Indian provider images had the highest proportion of fashion-related labels due to elements like feathers and jewelry, while Pacific Islander images had notably fewer medical and formal clothing labels, reinforcing traditional and less professional stereotypes. These findings highlight the ethical implications of AI-driven representations that can perpetuate stereotypes that may impact public perceptions of healthcare professionals from underrepresented groups.

### 4.1 Limitations and ethical considerations

We used DALL-E 3 to generate synthetic images, leveraging its extensive training dataset of approximately 1 billion images (7). However, the full details of this dataset are not publicly available, and the algorithm is regularly updated, which may impact consistency in future studies. However, the study's goal is to present a partially automated Generative AI/ML framework that can adapt to the evolving nature of these algorithms.

We used human scoring to quantify diversity scores, which is susceptible to individual biases, although minimized with consensus scoring and validated with DeepFace. The terms used in the study do not include multiracial individuals, although the race diversity scale accounts for multiracial appearing images. The racial categories used are limited to those defined by the U.S. Census Bureau. The measures of diversity assessed in this study focus on biological attributes; future iterations could explore the representation of socially derived attributes, such as gender identity and socioeconomic diversity, to provide a more comprehensive understanding of diversity in healthcare.

Finally, as this study proposes a framework that leverages Generative AI and ML, ethical concerns are important to address. The generated images may be derived from other available images on the Internet, which can be mitigated through greater transparency, such as openly documenting the datasets used in the framework. Additionally, the framework must be adapted periodically as Generative AI models are updated to reduce the risks of outdated insights. Any users of the framework must not misinterpret the results, requiring comprehensive guidelines to promote responsible use. Users should recognize that the diversity metrics used in the study are not comprehensive (for example, does not include socially derived attributes) to avoid conclusions based entirely on this framework.

## 5 Conclusions

In conclusion, our study introduces a ML-based methodology to quantify diversity and identify biased perceptions in AI-generated representations of healthcare providers. These stereotypes have real-world implications, as they undermine patient trust toward providers from underrepresented groups. This can lead to reduced diversity amongst healthcare professionals, negatively affecting patient care by limiting the range of perspectives and cultural competence that inform medical decision-making. Patients from underserved socioeconomic, racial, or gender backgrounds may be unable to access providers who understand their unique needs.

The study's approach offers valuable real-time insights for stakeholders such as medical policymakers seeking to address and monitor biases within AI tools used by the healthcare workforce. It can also inform training materials for healthcare professionals by guiding representations that are less stereotyped and biased. The study's methodology is not limited to healthcare, including education, media, politics, and entertainment. As these contexts often serve or appeal to a diverse audience, the framework can be useful for evaluating sources of bias.

## Data Availability

The original contributions presented in the study are included in the article/Supplementary Material, further inquiries can be directed to the corresponding author.
