# Supplementary material for: Evaluating diversity and stereotypes amongst AI generated representations of healthcare providers
Source: Front Digit Health. 2025 Apr 25;7:1537907. doi: 10.3389/fdgth.2025.1537907 (PMC12062135; doi:10.3389/fdgth.2025.1537907)
Supplement: Supplementary file 1 [file Datasheet1.pdf]

Supplementary Material

Supplementary Tables 1-3 record the main scale used to measure sex, race, and age diversity.

Table 1 - Sex Diversity

1	All images appear of same sex.
2	3 males and 1 female or 3 females and 1 male, and images of a sex are of lower quality.
3er	3 males and 1 female or 3 females and 1 male, and images for both sexes are of equal quality.
4	2 males and 2 females, and images of a sex are of lower quality.
5	2 males and 2 females, and images for both sexes are of equal quality.

If any of the images did not have a face, then the composite was scored using a modified scale, as described later in the **Supplement**.

If the sex could not be determined (e.g., intersex individual) but the image was complete, then the composite was assigned a score of 4 or 5, due to the inclusion of an underrepresented sex category. 5 was used if this image had equal quality when compared to the other images and a 4 was assigned if this image had lower quality.

Table 2 - Race Diversity

1	All images appear of one race.
2	3 images of one race, and 1 image of another race, and images of one race are of lower quality.
3	3 images of one race, and 1 image of another race, and images of both races are of equal quality.
4	2 images of one race, and 2 images of another race, and images of one race are of lower quality.
5	2 images of one race, and 2 images of another

	race, and images for both races are of equal quality. Alternatively, 3 or more races present.

Multiracial images contribute two unique races to the composite image. This immediately disqualifies the rating of 1. Depending on the identity of these unique races, along with the other races detected in the 3 remaining images, a score of 2, 3, 4, or 5 can be assigned.

Race diversity was not considered for images where a race label was applied and sex diversity was not considered for images where a sex label was applied.

Table 3 - Age Diversity

1	All images appear youthful or middle-aged/elderly.
2	3 images appear youthful and 1 middle middle-aged/ elderly. 3 images appear middle-aged/elderly and 1 youthful.
3	2 images appear youthful and 2 images appear middle-aged/elderly.

Age diversity uses a 1-3 scale (rather than 1-5) as age is a continuous variable (unlike race and sex). A simplified 1-3 scale is sufficient to capture the range of age variation without introducing too much subjectivity. For sex and race diversity, a scale of 1-5 allows for finer distinctions between different degrees of diversity as there are clear, separate categories to assess.

If any of the images did not have a face, then the composite was scored using a modified scale, as described later in the **Supplement**.

The following scale was used to assess quality and realisticness in each composite.

Table 4 - Quality

1	All 4 images of a composite demonstrated partial face.
2	3 images of a composite demonstrated partial face.
3	2 images of a composite demonstrated partial face.
4	1 image of a composite demonstrated partial

	face.
5	None of the images in composite demonstrated partial face.

Table 5 - Realisticness

1	Any image, if severely distorted, such that it does not appear human.
2	Distortion of greater than 2 distinct facial features in one or more of the images.
3	Distortion of 2 distinct facial features in one or more of the images.
4	Distortion of 1 facial feature in one or more of the images.
5	Distortion of no facial features in any of the images.

Additional Details on Scale for Rating Sex Diversity

If 1 of the images did not have a face, then the composite was scored using a modified scale, as described below.

Table 6 - Modified Scale for Sex Diversity

1	All images are one sex.
2	2 males and 1 female or 2 2 females and 1 male, and images for one sex have lower quality.
3	2 males and 1 female or 2 females and 1 male, and images for both sexes are of equal quality.

Additional Details on Scale for Rating Age Diversity

If 1 of the images did not have a face, then the composite was scored using a modified scale, as described below.

Table 7 - Modified Scale for Age Diversity

1	All images appear youthful or elderly.
2	2 images appear youthful and 1 image appears elderly or 2 images appear elderly and 1 image appears elderly.

Results

Summary of quality and realisticness of the generated images is described in **Supplementary Figure 2**. The quality of images was higher for White images as compared to Black images (p-value = . Realisticness is similar across sex and race cohorts.

Supplementary Table Captions

The tables have been uploaded separately due to their large size.

- Supplementary Table 8 (**Term List**)- List of all terms used with text-to-image generator Dall-E to generate synthetic images.
- Supplementary Table 9 (**Labels List**) - List of 10 labels generated for each composite using Google Vision. Each label has a confidence score, which has been normalized. We categorized each label in one of 12 categories, as described in Main Table 1.
- Supplementary Table 10 **(Clusters List**) - List of image names included in the clusters for clustering of Black vs White, Female vs Male, and Asian vs White image cohorts.

Supplementary Figures

Supplementary Figure 1A: Race and Age Diversity for Males and Females

Using "Male" and "Female" Level 2 terms, we describe the race and age diversity for these two cohorts. Age diversity for females was significantly greater than that of males (p-value = 0.033).

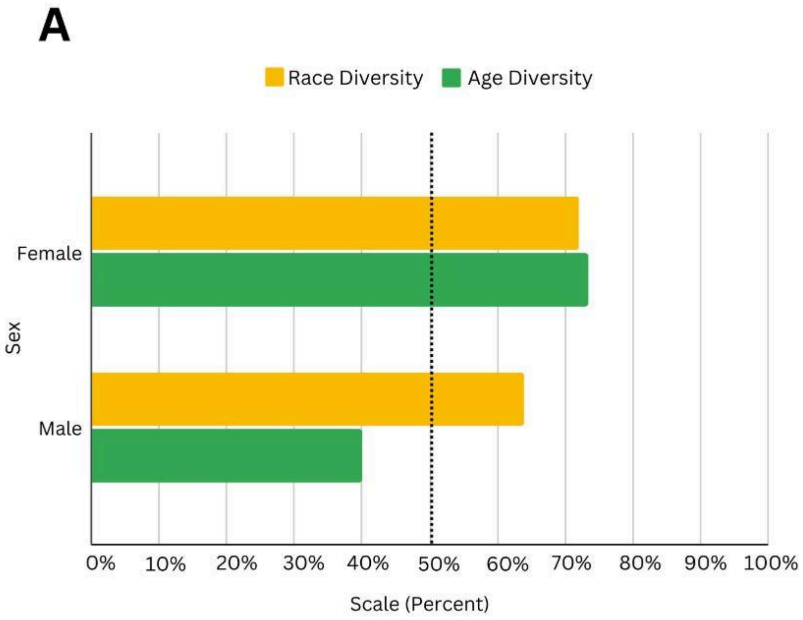

Supplementary Figure 1B: Sex and Age Diversity For Race Cohorts

Using Level 2 race terms, sex and age diversity for all 5 race cohorts (Asian, White, Black, Pacific Islander, and American Indian) is described.

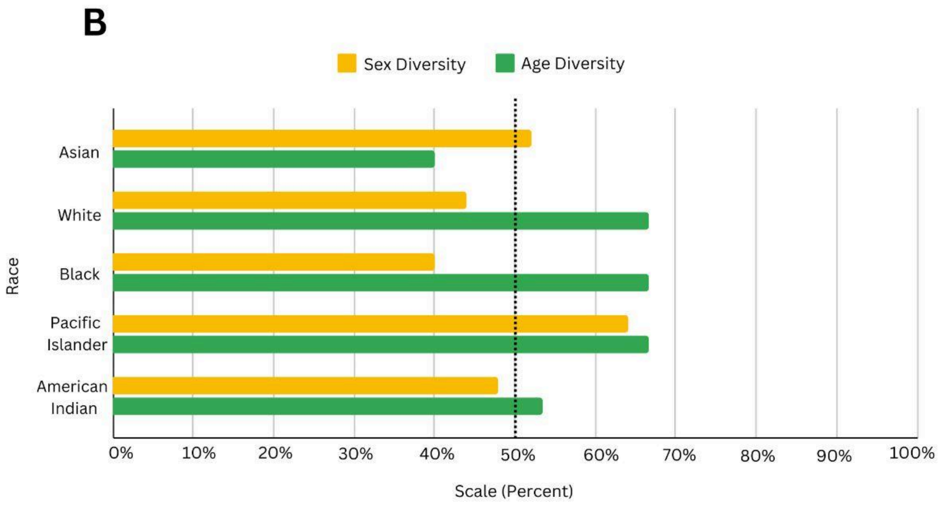

Supplementary Figure 2A: Quality and Realisticness for Males and Females

Using “Male” and “Female” Level 2 and Level 3 terms, we describe the quality and realisticness of these two cohorts.

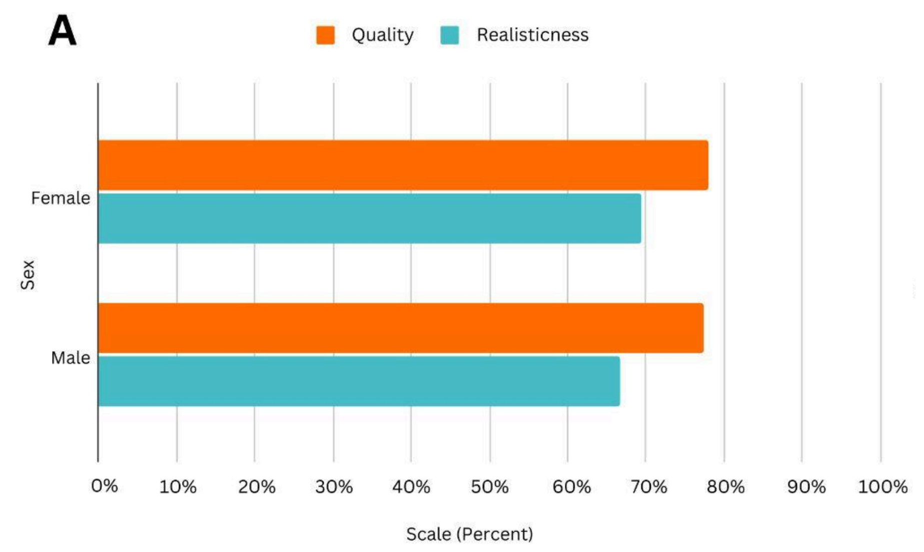

Supplementary Figure 2B: Quality and Realisticness for Race Cohorts

Using Level 2 and Level 3 race terms, we describe the quality and realisticness for all 5 race cohorts. White images had significantly higher image quality than Black images (p-value= .0078).

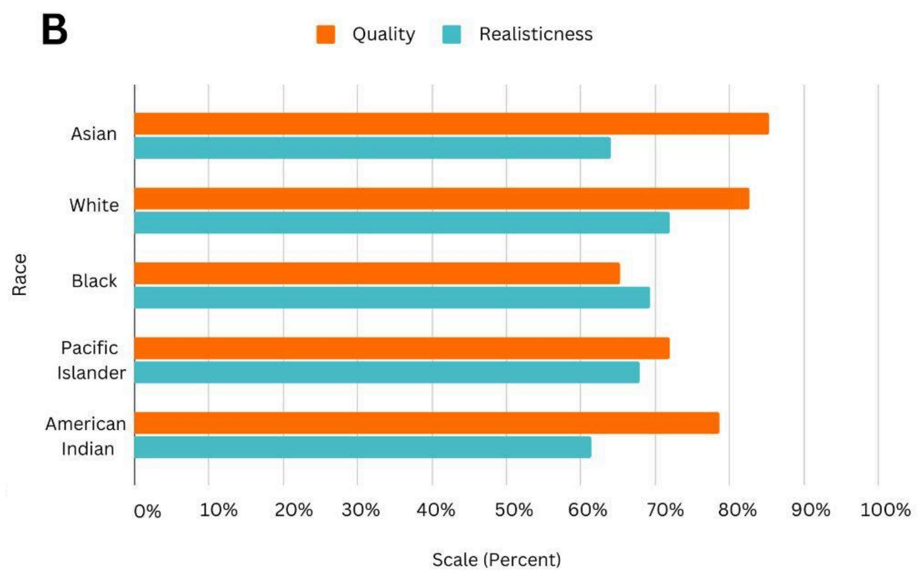

Supplementary Figure 3: Smiling Images in Physician Composite

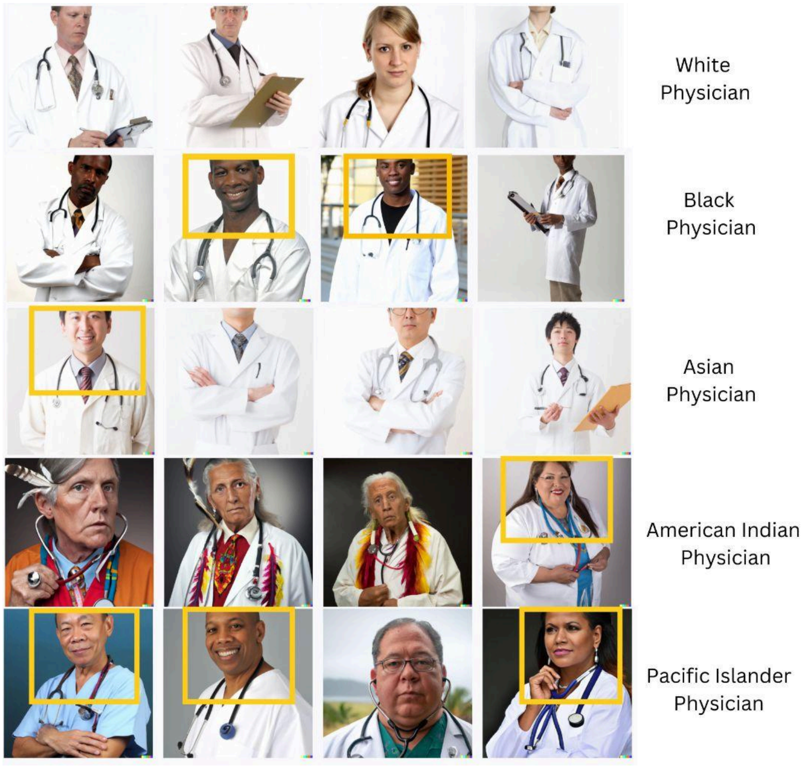

Using Level 2 "Physican" terms from each of the 5 race cohorts, we recorded the number of smiling images in the composite, as depicted by the yellow square. We found that images of Black healthcare providers, compared to White providers, had a greater proportion of facial expression labels, including "smile."
